# Brain gray matter changes in children at risk for sudden unexpected death in epilepsy

**DOI:** 10.1038/s41390-024-03295-0

**Published:** 2024-07-11

**Authors:** Bhaswati Roy, Jennifer A. Ogren, Luke A. Allen, Beate Diehl, Raman Sankar, Samden D. Lhatoo, Rajesh Kumar, Ronald M. Harper

**Affiliations:** 1 Department of Anesthesiology and Perioperative Medicine, University of California Los Angeles, Los Angeles, CA 90095 USA; 2 Department of Neurobiology, University of California at Los Angeles, Los Angeles, CA 90095 USA; 3 Department of Clinical and Experimental Epilepsy, University College London Institute of Neurology, London, UK; 4 Department of Neurology and Pediatrics, University of California Los Angeles, Los Angeles, CA 90095 USA; 5 Department of Neurology, McGovern Medical School, University of Texas Health Science Center at Houston, Houston, TX USA; 6 Brain Research Institute, University of California Los Angeles, Los Angeles, CA 90095 USA; 7 Department of Radiological Sciences, University of California Los Angeles, Los Angeles, CA 90095 USA; 8 Department of Bioengineering, University of California Los Angeles, Los Angeles, CA 90095 USA

## Abstract

**Background:**

Potential failing adult brain sites, stratified by risk, mediating Sudden Unexpected Death in Epilepsy (SUDEP) have been described, but are unknown in children.

**Methods:**

We examined regional brain volumes using T1-weighted MRI images in 21 children with epilepsy at high SUDEP risk and 62 healthy children, together with SUDEP risk scores, calculated from focal seizure frequency. Gray matter tissue type was partitioned, maps normalized, smoothed, and compared between groups (SPM12; ANCOVA; covariates, age, sex, and BMI). Partial correlations between regional volumes and seizure frequency were examined (SPM12, covariates, age, sex, and BMI); 67% were at high risk for SUDEP.

**Results:**

The cerebellar cortex, hippocampus, amygdala, putamen, cingulate, thalamus, and para-hippocampal gyrus showed increased gray matter volumes in epilepsy, and decreased volumes in the posterior thalamus, lingual gyrus, and temporal cortices. The cingulate, insula, and putamen showed significant positive relationships with focal seizure frequency indices using whole-brain voxel-by-voxel partial correlations. Tissue volume changes in selected sites differed in direction from adults; particularly, cerebellar sites, key for hypotensive recovery, increased rather than adult declines.

**Conclusion:**

The volume increases may represent expansion by inflammatory or other processes that, with sustained repetitive seizure discharge, lead to tissue volume declines described earlier in adults.

**Impact:**

- Children with epilepsy, who are at risk for Sudden Unexplained Death, show changes in brain volume that often differ in direction of change from adults at risk for SUDEP.
- Sites of volume change play significant roles in mediating breathing and blood pressure, and include areas that serve recovery from prolonged apnea and marked loss of blood pressure.
- The extent of volume changes correlated with focal seizure frequency.
- Although the underlying processes contributing to regional volume changes remain speculative, regions of tissue swelling in pediatric brain areas may represent transitory conditions that later lead to tissue loss in the adult condition.

## Introduction

Children with epilepsy have increased risk of sudden unexpected death compared with the general population, especially those children with a high incidence of focal seizures. The reported rates of sudden unexpected death in epilepsy (SUDEP) in children ranges from 0.22–1.11/1000 person-years,^1^ while the rates for adults are 1.2/1000 patient-years. A very limited literature suggests that children with complex epilepsy presentations and comorbid conditions are at higher risk.^2^ over those with generalized tonic-clonic seizures (GTCS), a principal risk factor for adults.^3^

Determination of processes that lead to SUDEP usually focus on loss of oxygenation, typically from sustained apnea, or a failure in brain perfusion, possibly from arrhythmia or pronounced decline in blood pressure.^4,5^ Description of the neural regulatory processes underlying either of those failing mechanisms in adults has outlined alterations in regional brain volumes in sites mediating these vital systems, or disruptions in brain tissue integrity that could compromise respiratory or cardiac functions.^6–8^ Those descriptions that stratify tissue changes by risk for SUDEP, however, almost exclusively derive from adult patients,^9^ and may not represent conditions in pediatric cases. The pediatric brain undergoes substantial regional, and especially, cortical developmental changes that can greatly differ from adult brain structures.^10,11^ Since these developing brain structures can exert significant influences on vital breathing and blood pressure functions necessary to maintain survival, it is essential to determine the sites and extent of regional brain structural changes in children with epilepsy and to relate the extent of those changes to SUDEP risk.

The findings from adults indicated particular brain regions, such as areas within the cerebellum,^12^ showed major tissue changes in those who later succumbed to SUDEP, or who were at much higher risk for SUDEP. These brain areas are subjected to developmental changes, or are substantially influenced by, or exert influences on developing brain regions, *e.g*. in the case of cerebellar sites, to and from the frontal cortex. Several cerebellar areas are particularly important in the search for SUDEP processes, because of their role in mediating “last chance” restoration of blood pressure or termination of apnea.^13,14^ Other brain regions, such as the periaqueductal gray,^12^ were also altered in adults, and is a region essential in assisting respiratory activation; the periaqueductal gray receives input from the amygdala, which in turn has activity heavily influenced by cortical and limbic structures that undergo major developmental changes. Although the findings in adults are critical for understanding SUDEP processes, parallel evaluations in pediatric cases are lacking for examining structure or function in pediatrics for SUDEP risks.

Routine brain MRI and clinical evaluation, including T1-weighted imaging, typically does not yield sufficient evidence to support functional impairment in children with epilepsy. High-resolution T1-weighted imaging-based voxel-based morphometry (VBM), a whole-brain automated technique offers rapid, unbiased assessment of brain tissue on a voxel-by-voxel basis, and can show localized gray matter (GM) volume changes in pediatric epilepsy.^12^ The VBM procedures have been previously used in several conditions including obstructive sleep,^15^ heart failure,^16^ diabetes,^17^ and pulmonary hypertension^18^ to characterize brain structural changes.

Here, we aimed to examine regional GM volume changes in pediatric epilepsy patients, compared to healthy controls using VBM procedures. We hypothesized that children at high risk for SUDEP would show altered GM volume in cardiovascular and respiratory regulatory sites over healthy controls in a fashion similar to that found in adults, but more-restricted in extent of injury, reflecting shorter periods of excitotoxic and hypoxic injury exposure from that of adults, and that limbic areas normally underlying focal seizures may be more affected.

## Materials and methods

### Subjects

This study was a cross-sectional, comparative evaluation of twenty-one pediatric individuals with epilepsy (mean age ± SD, 14.1 ± 4.1 years; male, 9), recruited from the University of California Los Angeles (UCLA) pediatric epilepsy monitoring unit (EMU) and 62 control subjects (mean age ± SD, 16.0 ± 3.8 years; male, 35), recruited through advertisements at the UCLA campus and the Los Angeles area. Of 21 children with epilepsy, 11 pediatric individuals had nocturnal seizures, and 9 had intellectual disabilities. The etiologies of some cases were unknown, a few were due to genetic causes including heterozygous variant of SCN1A, ataxia with RARS2 mutation, and chromosome 3q28 deletion and trisomy 3 and trisomy 14, two due to left temporal cortical dysplasia, and one child had traumatic brain injury. All study procedures were followed in accordance with institutional guidelines, and the study was approved by the UCLA Institutional Review Board. All subjects and their parents/guardians gave informed written consent/assent before the study. The subjects with epilepsy were those with a GTCS captured during their non-invasive EMU admission. All subjects had peri-ictal pulse oximetry data collected around the time of GTCS (a window of at least 1 min prior to seizure onset, and 3 min post-ictal), and a T1-weighted MRI scan. The exclusion criteria were: incomplete physiological data (missing or incomplete pulse oximetry data), previous neurosurgery, large brain lesions, incomplete clinical data, or incomplete (or artifact-degraded) MRI scans. We excluded patients with known long-term history of phenytoin use, due to its association with brain structural changes. Control subjects were in good health, with no sleep disturbances, without any history of chronic medical or psychiatric conditions, or any previous history of head injury (e.g. concussions, trauma), and without neurological or cardiovascular disorders that could introduce brain tissue injury.

### Magnetic resonance imaging

All brain imaging studies were performed in a 1.5 or 3.0-Tesla MRI scanner [Magnetom Skyra (epilepsy, *n* = 4), Tim-Trio (epilepsy, *n* = 14; control *n* = 62), Prisma Fit (epilepsy, *n* = 1), and Signa HDxt (epilepsy, *n* = 2)]. We used foam pads on either side of the head to minimize head motion. High-resolution T1-weighted images were collected using a gradient-echo sequence (repetition time = 10–2200 ms; echo-time = 2.32–4.8 ms, flip angle (FA) = 8–25; number of averages = 1; matrix size = 256–320 × 192–320; field of view = 180–256 × 220–256 mm; slice thickness = 0.9–1.5 mm. We visually assessed images from all subjects for any major pathology, such as cystic lesions, infarcts, or tumors to subsequently exclude subjects if found with any abnormality. We critically examined all T1-weighted images for any head-motion related or other imaging artifacts as well.

### Data processing

The statistical parametric mapping package (SPM12, http://www.fil.ion.ucl.ac.uk/spm/), MRIcroN, and MATLAB-based (The MathWorks Inc., Natick, MA) custom software were used for data processing and analyses. T1-weighted image volumes were partitioned into gray matter, white matter, and cerebrospinal fluid (CSF) tissue types. The Diffeomorphic Anatomical Registration Through Exponentiated Lie algebra algorithm (DARTEL) toolbox was used to generate the flow fields, which are nonlinear deformations applied for warping all the gray matter images to match each other and template images that were implemented for normalization of gray matter maps to Montreal Neurological Institute (MNI) space (voxel size: 1 × 1 × 1 mm^3^). The modulated and normalized maps were smoothed using a Gaussian filter, and the smoothed gray matter maps were used for further statistical analyses.

### SUDEP risk evaluation

Focal seizure frequency rates were computed as the number of seizures occurring per day. A score of ≥13 focal seizures per year or a score of ≥3 GTCS per year was considered risk for SUDEP in children with epilepsy.^19^

### Statistical analyses

The Statistical Package for the Social Sciences (IBM SPSS, v28.0, Armonk, NY) was used for assessment of demographic and clinical variables using independent samples *t*-tests, and Chi-square tests (Table 1). A *p* value of <0.05 was considered statistically significant.

**Table 1:** Demographics and clinical characteristics of pediatric children and healthy controls.

Variables	Children with epilepsy *n* = 21 (Mean ± SD)	Controls *n* = 62 (Mean ± SD)	*p* values
Age (years)	14.1 ± 4.1	16.0 ± 3.8	0.06
Sex [male] (%)	9 (43%)	35 (56%)	0.28
BMI	20.8 ± 3.9	21.5 ± 4.4	0.54
Handedness [L/R/ambidex/undetermined]	[2/12/1/6]	[8/51/2/1]	0.002
Ethnicity	10 White, 5 Hispanic, 3 AAPI, 2 Black, 1 Other	–	–
Disease duration (years)	8.2 ± 5.2	–	
Seizures/per day	29.7 ± 108.3	–	–
SUDEP 7 scores	3.3 ± 1.9	–	–
Number AEds	2.7 ± 0.9	–	–

*SD* standard deviation, *BMI* body mass index, *L* left, *R* right, *SUDEP* sudden unexpected death in epilepsy, *AEDs* anti‐epileptic drugs.

**Fig. 1:**
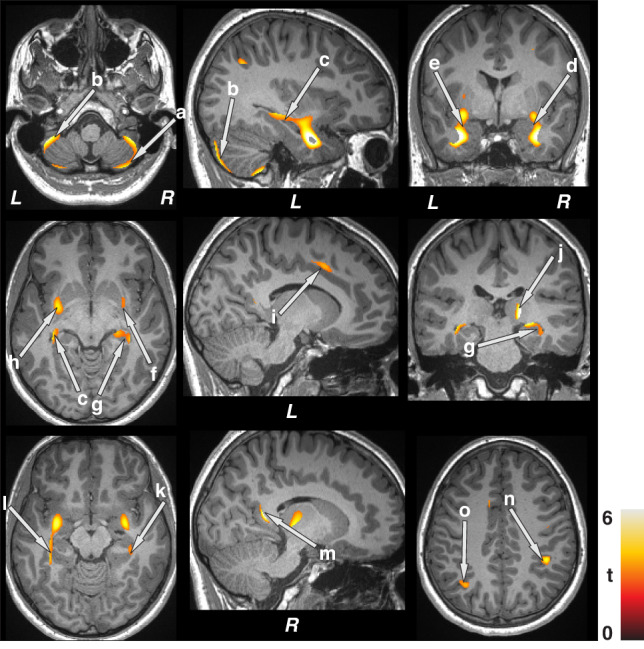
Brain regions with higher gray matter volume in children with epilepsy over health controls after controlling for age, sex, and BMI (FDR corrected, *p* < 0.05). These sites with increased gray matter volumes included the bilateral cerebellar cortex (a, b), hippocampus (c, g), amygdala (d, e), putamen (f, h), mid (i), and posterior (m) cingulate, right thalamus (j), bilateral para-hippocampal gyrus (k, l), and parietal cortices (n, o). All images are in neurological convention (L left, R right). Color bar indicates *t*-statistic values.

**Fig. 2:**
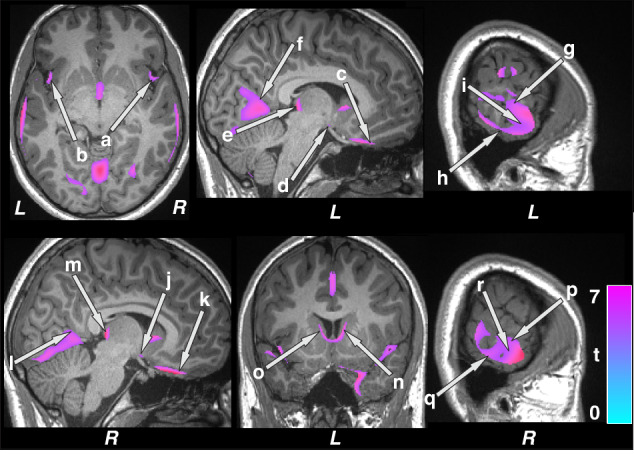
Brain sites with lower regional gray matter volume in patients with epilepsy compared to control subjects. Brain regions with reduced gray matter volumes were observed in the bilateral insula (a, b), ventral medial prefrontal cortices (c, k), hypothalamus (d, j), posterior thalamus (e, m), lingual gyrus (f, l), inferior (h, q), mid (i, r), and superior (g, p) temporal cortices, and caudate (n, o) in patients with epilepsy over controls. Figure conventions are the same as in Fig. 1.

For regional GM volume differences between groups, the smoothed whole-brain GM maps of children with epilepsy and controls were compared using analysis of covariance (ANCOVA), with age, sex, and BMI as covariates [SPM12; *p* < 0.05, false discovery rate (FDR) correction for multiple comparisons; Figs. 1 and 2]. The statistical parametric maps showing brain sites with significant GM volume differences between groups were superimposed onto an anatomical image, created from a control subject, for anatomical identifications. Additional analyses were performed to evaluate regional GM volume differences between children with epilepsy and controls using ANCOVA [covariates, age, sex, BMI, symptomatic epilepsy, nocturnal seizures, and neurodevelopmental disabilities, uncorrected *p* < 0.005] (Supplementary Figs. 1 and 2).

**Fig. 3:**
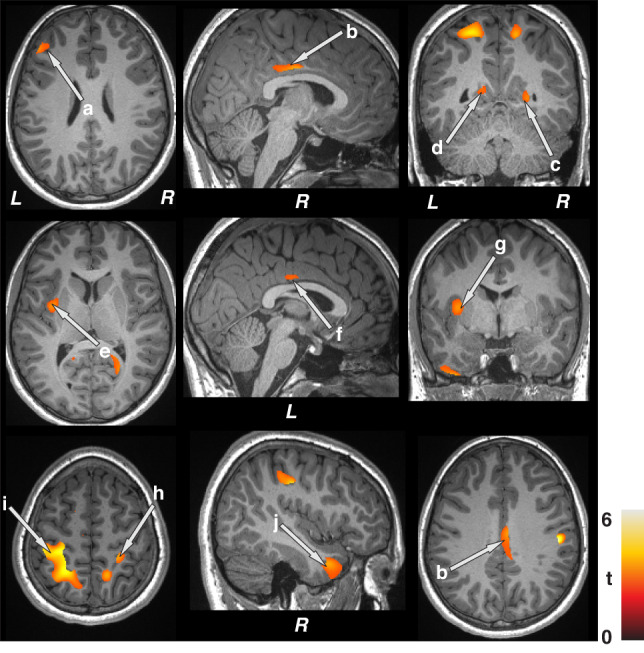
Associations observed between gray matter volumes and focal seizure frequency indices using whole-brain voxel-by-voxel partial correlation procedures (covariates; age, sex, and BMI). Positive correlations emerged between gray matter volumes and focal seizure frequency indices in children with epilepsy in the frontal cortices (a), bilateral mid (b, f) and posterior (c, d) cingulate, left insula (e), putamen (g), bilateral parietal cortices (h, i), and temporal cortices (j). Figure conventions are the same as in Fig. 1.

Whole-brain GM volume maps were correlated voxel-by-voxel with focal seizure frequency index (number of seizures per day) in epilepsy patients using partial correlations (SPM12; covariates, age, sex, and BMI, uncorrected, *p* < 0.005, minimum extended cluster size, 40 voxels) (Fig. 3). Brain clusters showing significant correlations between gray matter volumes and focal seizure frequency indices were overlaid onto background images for structural identification. Additionally, partial correlations were performed with covariates, age, sex, BMI, symptomatic epilepsy, nocturnal seizures, and neurodevelopmental disabilities (SPM12; uncorrected, *p* < 0.005, minimum extended cluster size, 40 voxels) (Supplementary Fig. 3).

## Results

### Demographic and clinical characteristics

Demographic and clinical variables of patients with epilepsy and control subjects are summarized in Table 1. No significant differences in age (*p* = 0.06), sex (*p* = 0.28), or BMI (*p* = 0.54) appeared between groups. Of 21 epilepsy patients, 14 patients (66.7%), based on focal seizures or 7 children (33.3%), based on GTCs were considered at high-risk for SUDEP (Table 1).

### Regional gray matter volume changes

Multiple brain areas showed increased regional gray matter volume in children with epilepsy compared to control subjects (Fig. 1; covariates, age, sex, and BMI), including the bilateral cerebellar cortex (a, b), hippocampus (c, g), amygdala (d, e), putamen (f, h), mid (i), and posterior (m) cingulate, right thalamus (j), bilateral para-hippocampal gyrus (k, l), and parietal cortices (n, o). Decreased volumes were prominent in more-rostral regions that serve prominent roles in CO_2_ and O_2_ integration, including the posterior thalamus (Fig. 2 e, m), and blood pressure regulation, including the bilateral ventral medial frontal cortices (c, k), bilateral insula (a, b) and hypothalamus (d, j). Decreased gray matter volumes also appeared in inferior (h, q), mid (i, r), and superior (g, p) temporal cortices, lingual gyrus (f, l), and caudate (n, o) in pediatric patients with epilepsy compared to controls (Fig. 2). Similar regions emerged showing increased gray matter volumes in children with epilepsy compared to controls after controlling for additional covariates including symptomatic epilepsy, nocturnal seizures, and neurodevelopmental disabilities (Supplementary Fig. 1). However, fewer sites with decreased gray matter volumes appeared in children with epilepsy compared to healthy controls after accounting for additional covariates (Supplementary Fig. 2).

### Associations between gray matter volumes and focal seizure frequency indices

Focal seizure frequency indices showed positive correlations with regional gray matter volumes in multiple areas (Fig. 3) using whole-brain voxel-by-voxel partial correlation procedures. These regions included the frontal cortices (a), bilateral mid (b, f) and posterior (c, d) cingulate, left insula (e), putamen (g), bilateral parietal cortices (h, i), and temporal cortices (j) in those children at high risk for SUDEP. The correlation coefficients demonstrating the strength of the associations between gray matter volume abnormalities and focal seizure frequency indices are shown in Table 2. These regions survived significance levels even after adding additional covariates, such as symptomatic epilepsy, nocturnal seizures, and neurodevelopmental disabilities (Supplementary Fig. 3).

**Table 2:** Correlation between regional gray matter volume and focal seizure frequency indices in children with epilepsy.

Brain regions	Correlation coefficient	*p* values
Left putamen	0.62	0.008
Left anterior insula	0.64	0.005
Right lingual gyrus	0.65	0.005
Left mid cingulate	0.63	0.007
Right mid cingulate	0.65	0.004
Left posterior cingulate	0.62	0.009
Right posterior cingulate	0.66	0.004
Left inferior temporal cor	0.64	0.006
Left mid temporal cor	0.62	0.008
Right mid temporal cor	0.62	0.008
Left superior parietal cor	0.71	0.002
Right superior parietal cor	0.68	0.003
Left mid frontal cor	0.64	0.006
Left superior frontal cor	0.64	0.006
Right superior frontal cor	0.63	0.007
Left precentral gyrus	0.71	0.002
Left postcentral gyrus	0.75	<0.001
Right postcentral gyrus	0.74	<0.001

*Cor* cortices, *Mid* middle.

## Discussion

These data suggest that children with epilepsy, the majority of whom were at high risk of SUDEP, have altered gray matter volumes, with strong associations between gray matter volume abnormalities and focal seizure frequency indices. Gray matter volume increases in children with epilepsy appeared in widespread areas, and included the cerebellum, hippocampus, amygdala, putamen, cingulate, thalamus, para-hippocampal gyrus, and parietal cortices. Gray matter volume loss emerged in the insula, ventral prefrontal cortices, lingual gyrus, caudate, and temporal cortices in children with epilepsy (covariates, age, sex, and BMI). However, after accounting for additional covariates including symptomatic epilepsy, nocturnal seizures, and neurodevelopmental disabilities, fewer sites showed reduced gray matter volumes in children with epilepsy compared to controls. Correlational analyses showed associations between SUDEP risk factors, including focal seizure frequency indices, and gray matter volume integrity in multiple brain sites, including the frontal cortices, cingulate, insula, putamen, parietal, and temporal cortices.

Previous studies on adult patients with epilepsy at risk for SUDEP showed increased gray matter volumes in multiple limbic areas, including the amygdala, hippocampus, entorhinal and para-hippocampal cortex, and subcallosal cortex,^12,20–22^ but significant tissue loss in the cerebellum, periaqueductal gray, left posterior and medial thalamus, left hippocampus, and posterior cingulate.^12^ The cerebellar loss in adults at risk for SUDEP was a concern, since the medial accessory olive receives projections from the nucleus of the solitary tract (NTS), and then projects to the Purkinje cells of the cerebellar vermis, which, in turn, send fibers to the fastigial deep cerebellar nuclei, and form a protective recovery network to restore blood pressure and restart breathing from extreme hypotension or apnea.^13,14,23,24^ Restoration of blood pressure is a major concern in SUDEP, and absence of respiratory efforts contributes to a loss of return to normotensive levels. Injury to the fastigial nuclei also will impede recovery from sustained apnea.^14^ The fastigial nuclei contain chemosensitive neurons, and injury attenuates respiratory responses to even modest levels of hypercapnia or hypoxia, a critical issue for recovery from post-ictal apnea accompanying SUDEP. Damage to components of this circuitry, especially to the fastigial nuclei found in adult cases who succumbed or were at high risk for SUDEP,^12^ are of particular interest in adults with epilepsy.

The cerebellar volume increases in children with epilepsy especially differed from the losses found in adults. In addition to the volume increase vs. decrease, no major changes were found in the deep cerebellar nuclei in the pediatric cases. The most likely interpretation of the adult/pediatric differences is that the pronounced tissue loss in adults results from prolonged progressive injury, while the tissue volume increases in children represent transitional states with inflammatory swelling. Such an interpretation could be resolved with histological studies. Specific histological changes are just now being described, most recently in the amygdala,^20,21^ and help to provide a basis for the tissue volume increases in adults, but comparable studies need to be performed in children at risk for SUDEP. It should be noted that the volume increase in pediatric cerebellar cortex, if representing inflammatory processes, still represents a condition in which functions of the brain site may be compromised. Increased amygdala volume in adults at risk for SUDEP, for example, show misformed microstructure.^20^ that almost certainly would contribute to impaired function.

A few postmortem studies^25,26^ on a small number of adult patients with generalized epilepsy suggest microdysgenesis and increased neuronal densities, leading to diminished gray-white matter demarcation, and increased gray matter volumes. The blurring of the gray-white matter interface might result from disorganization of the gray matter ribbon at the microscopic level, dystopic neurons in adjacent white matter sites, or abnormal neurons affecting image signal intensity.^27^ Novel MRI procedures, such as neurite orientation dispersion and density imaging (NODDI), allow a non-invasive means to evaluate histological changes in children and represent a goal to understand the tissue volume changes.^20^

Selected sites showed increased gray matter volume in previous studies on adult patients with epilepsy at risk for SUDEP; these sites included the amygdala and nearby temporal cortex,^12,20–22^ and increased volumes in those structures also appeared in children. Another postmortem study in infants who succumbed to SUDEP.^28^ showed abnormalities in the hippocampus. Increased gray matter volume in the hippocampus and amygdala might be indicative of gliosis.^29,30^ and plasticity following neural injury, and may include neuronal and synaptic functional alterations that have been associated with excitotoxic injury in epilepsy.^31^

Of particular interest was the finding of diminished gray matter volumes in areas generally recognized as critical for blood pressure control or for integration of CO_2_ and hypoxia. Roles for the insula in mediating both sympathetic (principally right insula) and parasympathetic (mainly left insula) drives to modulate blood pressure are well described; both left and right insulae were affected here.^32–34^ Similarly, the ventral medial prefrontal cortex bilaterally showed tissue volume decline; that cortical region, together with the insula, hypothalamus, and hippocampus are critical components of forebrain circuitry necessary for blood pressure maintenance.^35,36^ The hippocampus, as well as the amygdalae showed increased gray matter volume, indicating likely pathology in structure that would lead to dysfunction. The decline in volume in components of the principal circuitry for blood pressure control on the rostral brain is a concern in SUDEP. Those deficits, in combination with the increased volumes in cerebellar cortex, also likely a sign of abnormal structure and function, raise the possibility for compromised blood pressure regulation in these cases.

Developmental ventilatory studies in lambs outlined the critical role for regions within the posterior thalamus in O_2_ regulation in early life,^37^ and functional MRI studies in congenital central hypoventilation patients have shown that the posterior thalamus contributes to integration of both low oxygen and hypercarbia.^38,39^ A failure to appropriately integrate low oxygen or high CO_2_ during the post-ictal apnea period would pose a significant concern for survival.

Reciprocal networks between thalamic and cortical pyramidal neurons play a central role in generalized spike-wave discharge generation in patients with epilepsy;^40^ increased gray matter volume appeared in the right thalamus, and volume loss emerged in the bilateral posterior thalamus. The basis for aberrant conduction of discharge signals appears to be established already in these children. Abnormalities in the caudate and prefrontal cortices have been reported earlier in adult epilepsy patients,^41^ accompanied by poor performance on working memory tasks, since these regions play crucial role in visual working and prospective memory and executive functions. The findings of abnormal tissue volumes in these areas in children suggest the potential for comparable performance concerns.

Adults at high-risk of SUDEP showed functional connectivity differences between autonomic regulatory brain regions, including the thalamus, cingulate, putamen, and amygdala compared to those epilepsy patients with low-risk SUDEP.^42^ Comparable connectivity data for pediatric cases are not available; such data would be valuable to determine any functional changes in interactions between our structural findings of reduced volume in the prefrontal ventral medial cortex and other limbic structures in regulation of blood pressure.

Although extensive descriptions of neuroimaging findings exist for adult epilepsy patients at high-risk for SUDEP, and some of the adult findings are shared with pediatric cases, distinct differences emerged both in extent of affected tissue and in direction of volume changes. Thus, the adult findings cannot be simply generalized/extrapolated to children and adolescents. The separate outcomes likely stem from the differences in pathophysiology, severity, course, treatment, and response to treatment between adults and pediatric cases. However, comparisons may provide insights into the developmental course of tissue pathology in epilepsy.

Brain regions regulating crucial functions, dysfunctions of which create relevant issues in the circumstances surrounding SUDEP, showed altered volumes in our study. The hippocampus, a potential central modulator of augmented breaths due to its involvement in the emotional expression of stress, anxiety, and defensive emotional postures, behaviors known to affect the frequency of augmented breaths,^43,44^ showed increased volumes here. Single pulse stimulation of the amygdala can pace inspiration,^45^ while train stimulation can induce apnea.^46^ during seizures in children with epilepsy, and may play a critical role in cessation of breathing during and following ictal events.

Adult epilepsy patients with high risk of SUDEP showed increased amygdala volumes and lower neurite density indices, which represents the packing density of neurites, suggesting disruption in the nuclear organization influencing descending inputs to cardiovascular and respiratory regulatory sites, contributing to impaired control of blood pressure and respiratory timing, and thus, increasing the risk for sudden death.^20^ A comparable analysis of microstructure needs to be performed in children with epilepsy.

The putamen and thalamus showed increased volumes in children with epilepsy, which play a significant role in gating respiratory information between the cortex and the brainstem,^47^ and regulation of autonomic activity in the basal ganglia. The insula and cingulate, regions with altered volume (covariates: age, sex, and BMI) here are responsive to respiratory challenges, including hypercapnia, low tidal volume, and respiratory loads;^48–51^ deficiencies arising from conditions inducing the volume change could affect vital functions essential to recover from a challenge that might lead to SUDEP.

### Limitations

Although we collected ethnicity data from all the children with epilepsy, we do not have information related to ethnicity from controls, an issue that is a limitation of this study. Most of the patients with epilepsy and control children were scanned at a Siemen’s Tim-Trio scanner; seven patients were scanned on a different scanner. We scanned four control subjects in both Tim-Trio and Prisma scanners to study histogram changes of intensity of normalized, smoothed, and descalped gray matter volume maps, and found no differences in distribution of gray matter volume from two different scanners (Supplementary Fig. 4). Furthermore, we also plotted histograms of all patients with epilepsy from different scanners and all control children (Supplementary Fig. 5) and found that the histogram patterns were similar. However, use of different scanners can be considered a limitation. All the analyses accounted for covariates age, sex, and BMI, and further analyses (Supplementary figures) were performed with additional covariates, such as symptomatic epilepsy, nocturnal seizures, and neurodevelopmental disabilities; similar findings were observed, except for decreased gray matter in pediatric patients with epilepsy compared to controls. Very few regions survived showing decreased gray matter after the additional covariates were added owing to the small sample size of pediatric epilepsy patients. Thus, the results should be interpreted cautiously.

## Conclusions

Children with epilepsy, the majority of whom were at high risk of SUDEP, showed regional brain volume changes compared to healthy children in sites that are crucial for a range of functions, including breathing and blood pressure control. Brain regions with increased gray matter volumes emerged in the cerebellar cortex, hippocampus, amygdala, putamen, cingulate, thalamus, while reduced gray matter volumes appeared in the insula, temporal, prefrontal, and caudate regions in children with epilepsy after controlling for age, sex, and BMI; the structures with reduced volumes are especially involved with cardiovascular or autonomic regulation. Some of these areas, such as the cerebellum and left hippocampus, differed in direction from the volume findings in adults, and volume changes in the cerebellum were substantially less extensive than those in adults. Most of the brain regions showing decreased gray matter volumes, however, did not survive after inclusion of additional covariates (symptomatic epilepsy, nocturnal seizures, and neurodevelopmental disabilities). Several brain areas showed positive correlations on whole-brain voxel-by-voxel partial correlation analyses between gray matter volume and focal seizure frequency indices in children with epilepsy; these sites included frontal, cingulate, insula, putamen, parietal, and temporal areas. The processes contributing to volume increases and decreases, as well as to the volume differences with adult patients with epilepsy remain speculative, although consequences of excitotoxic and inflammatory mechanisms can be hypothesized. The increased volumes in defined areas in the pediatric condition, with reduced volumes in adults, suggests transitory processes are operating.

## Supplementary information

Supplementary Figures

## Data Availability

Since the data contain potentially sensitive personal health information on individual subjects, we are bound by ethical and legal restrictions on freely sharing data publicly. However, data from this study are available with approval from the UCLA Institutional Review Board for researchers who meet the criteria for access to confidential data.
