# Supplementary material for: Brain gray matter changes in children at risk for sudden unexpected death in epilepsy
Source: Pediatr Res. 2024 Jul 11;96(7):1732–8. doi: 10.1038/s41390-024-03295-0 (PMC11772226; doi:10.1038/s41390-024-03295-0)

Supplementary Fig 1: Brain regions with higher gray matter volume in children with epilepsy over healthy controls after controlling for age, sex, BMI, symptomatic epilepsy, nocturnal seizures, and neurodevelopmental disabilities ($p < 0.005$). These sites with increased gray matter volumes included the bilateral cerebellar cortex (a, b), hippocampus (c, g), amygdala (d, e), putamen (f, h), mid (i), and posterior (m) cingulate, right thalamus (j), bilateral para-hippocampal gyrus (k, l), and parietal cortices (n, o). All images are in neurological convention (L = left; R = right). Color bar indicates t-statistic values.

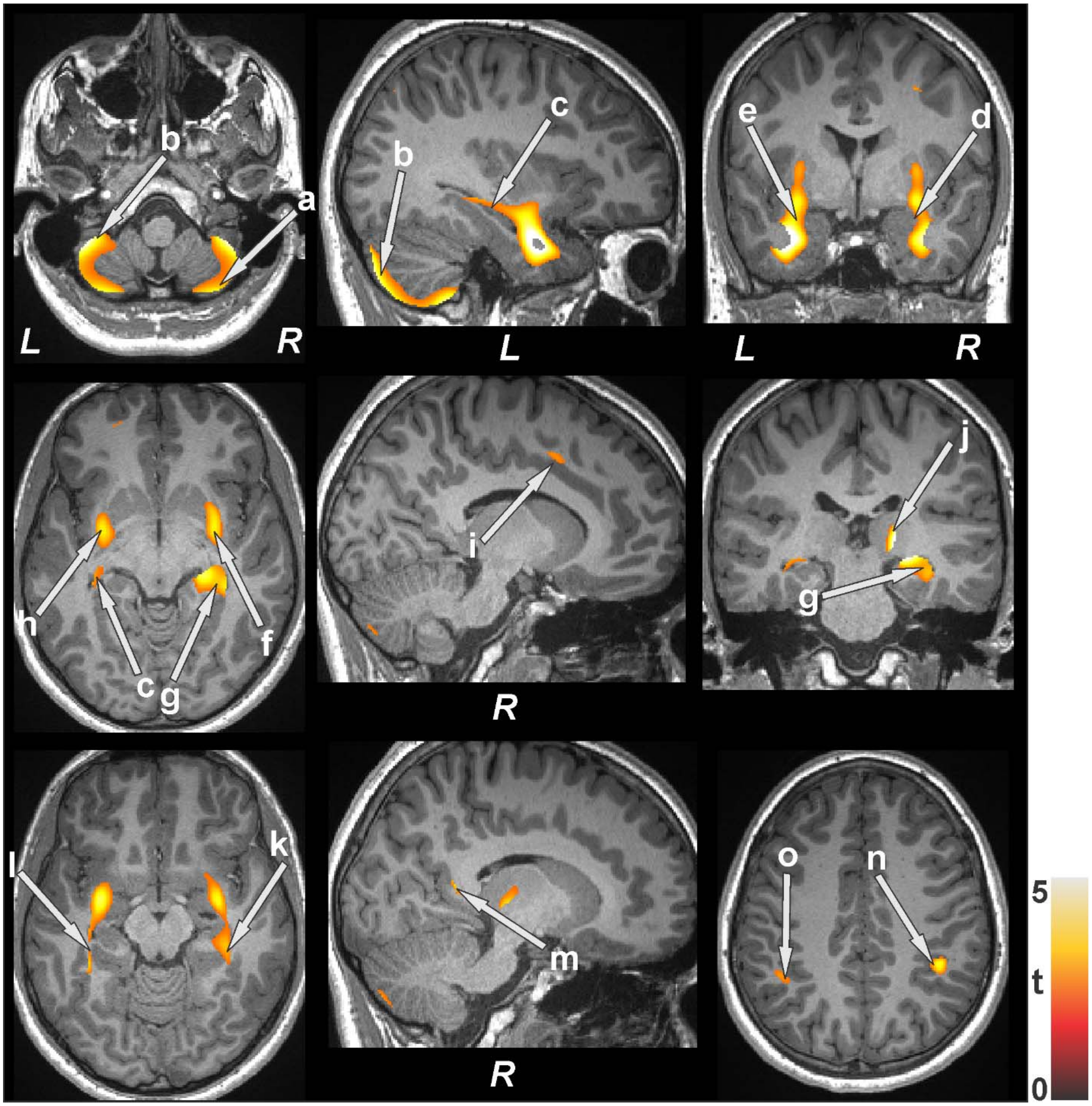

Supplementary Fig 2: Brain sites with lower regional gray matter volume in patients with epilepsy compared to control subjects after controlling for age, sex, BMI, symptomatic epilepsy, nocturnal seizures, and neurodevelopmental disabilities. Brain regions with reduced gray matter volumes were observed in the posterior thalamus (a, d), lingual gyrus (b, e), and mid (c, g) and superior (f) temporal cortices in patients with epilepsy over controls. Figure conventions are the same as in Fig. 1.

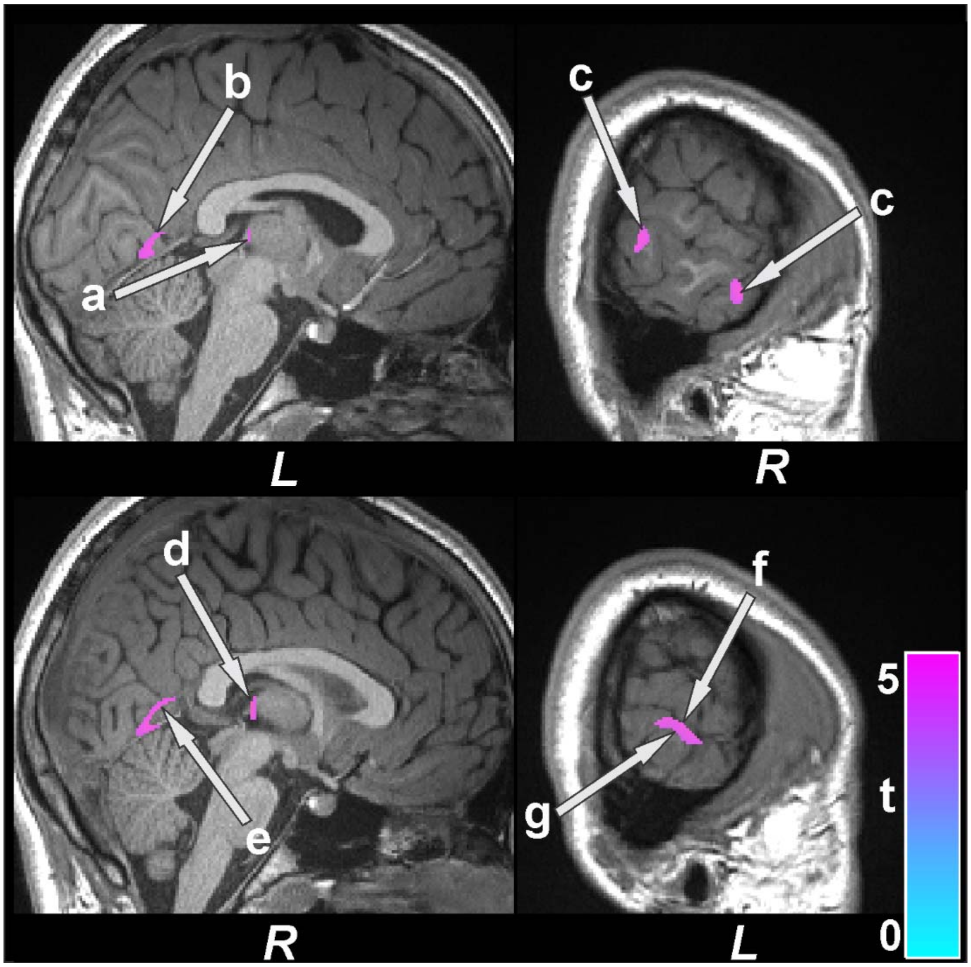

Supplementary Fig 3: Positive correlations emerged between gray matter volumes and focal seizure frequency indices in children with epilepsy after controlling for age, sex, BMI, symptomatic epilepsy, nocturnal seizures, and neurodevelopmental disabilities. These sites included the frontal cortices (a), bilateral mid (b, f) and posterior (c, d) cingulate, left insula (e), putamen (g), bilateral parietal cortices (h, i), and temporal cortices (j). Figure conventions are the same as in Fig. 1.

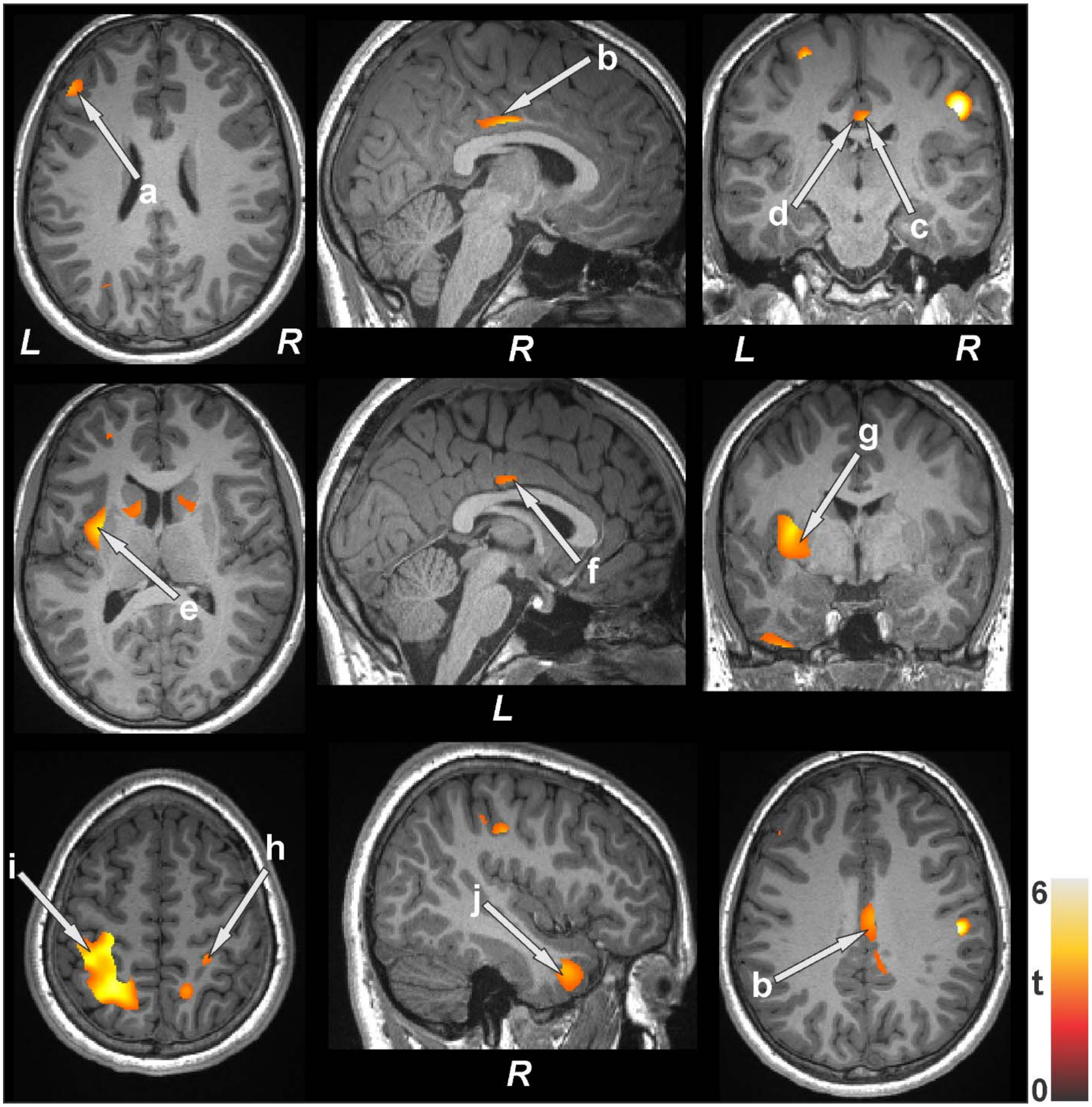

Supplementary Fig 4: Gray matter volume histograms of four control subjects scanned on two different scanners (3.0-Tesla, Magnetom, Tim-Trio, and Prisma). Gray matter volume histograms were plotted for normalized, smoothed, and descalped maps obtained from Tim-Trio (green) and Prisma (red) scanners. The x-axis (intensity) represents the bins for gray matter volume values. The Wilcoxon rank-sum test (Subject 1, p=0.60; Subject 2, p=0.87; Subject 3, p=0.83, Subject 4, p=0.33) showed no significant differences between histograms.

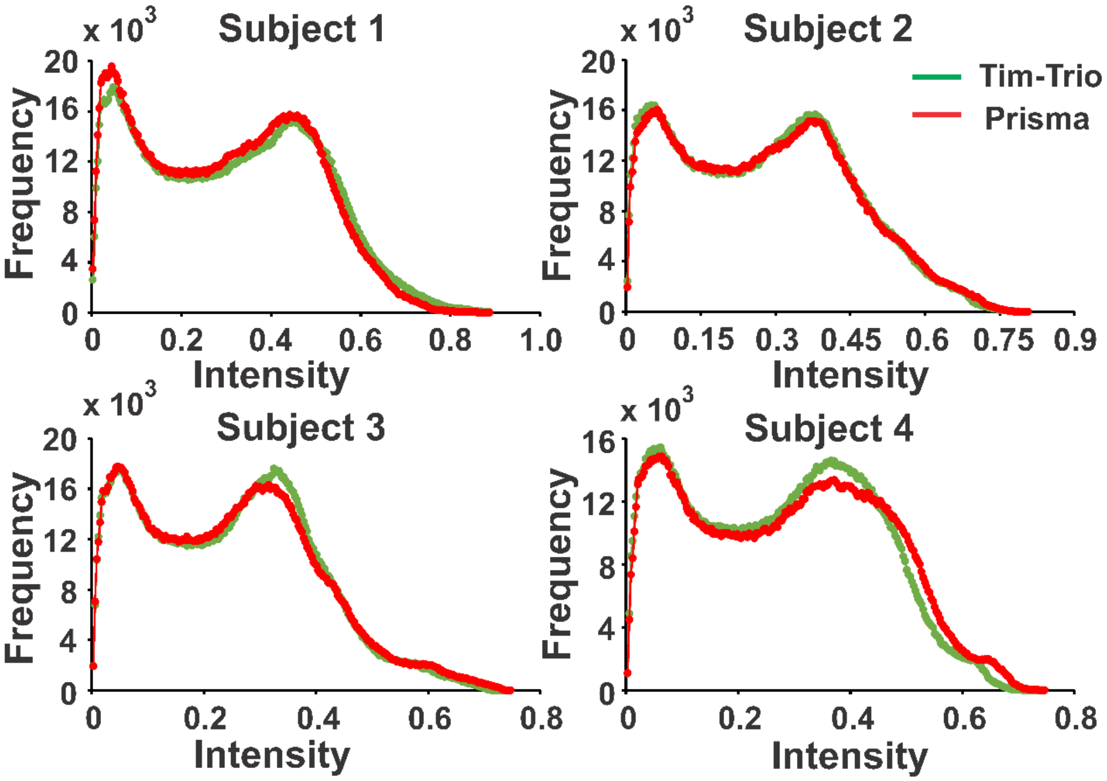

Supplementary Fig 5: Histogram plots of all the children with epilepsy and controls.

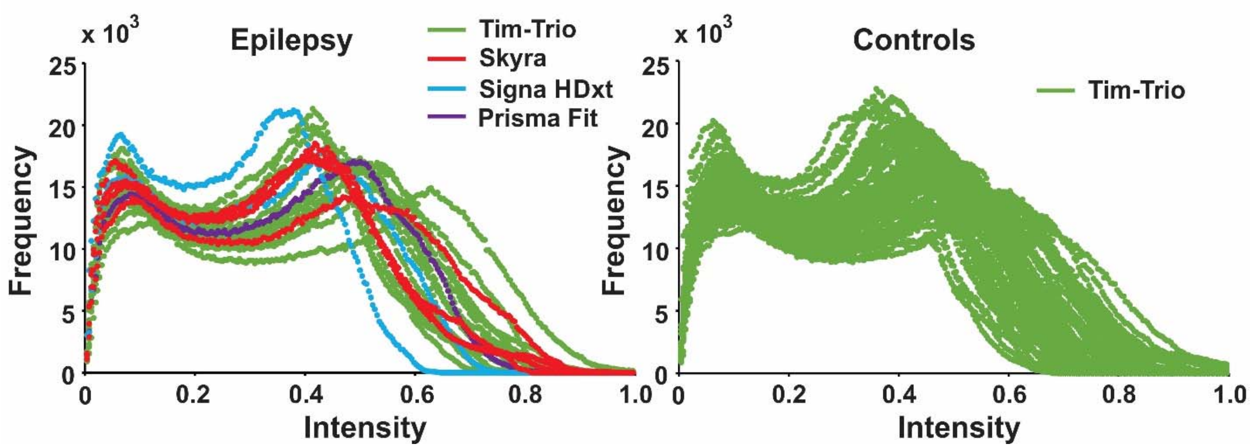

Supplement: Supplementary file 1 — Supplementary Figures [file 41390_2024_3295_MOESM1_ESM.pdf]
